# Antenatal iron supplementation and birth weight in conditions of high exposure to infectious diseases

**DOI:** 10.1186/s12916-019-1375-9

**Published:** 2019-07-26

**Authors:** Hans Verhoef, Martin N. Mwangi, Carla Cerami, Andrew M. Prentice

**Affiliations:** 1 Division of Human Nutrition and Health, Wageningen University, P.O. Box 17, 6700AA Wageningen, The Netherlands; 2 Cell Biology and Immunology Group, Wageningen University and Research, P.O. Box 338, 6700AH Wageningen, The Netherlands; 3 MRC Unit The Gambia at London School of Hygiene and Tropical Medicine, Atlantic Boulevard, Fajara, Banjul, The Gambia; 4 Training and Research Unit of Excellence, Department of Public Health, College of Medicine, University of Malawi, Private Bag 360, Chichiri, Blantyre 3, Malawi

**Keywords:** Anaemia, Birthweight, Iron, Malaria, Plasmodium, Pregnancy

## Abstract

**Background:**

A recent cohort study among Papua New Guinean women surprisingly showed iron deficiency during pregnancy to be associated with increased birth weight. These findings seemingly contradict previous trial evidence that iron supplementation leads to increased birth weight, particularly in iron-deficient women, and hence require explanation.

**Main text:**

We have re-analysed data from a previous trial in Kenya and demonstrated that, because women who were initially iron deficient respond better to iron supplementation, they show an increase in birthweight. There is evidence that this benefit is decreased in iron-replete women, possibly due to the adverse effects of haemoconcentration that can impair oxygen and nutrient transfer across the placenta. The Papua New Guinean results might be explained by a similar differential response to the iron supplements that they all received.

**Conclusions:**

Antenatal iron supplementation should ideally be administered in conjunction with measures to prevent, diagnose and treat malaria given the propensity of pathogenic microorganisms to proliferate in iron-supplemented individuals. However, even where services to prevent and treat malaria are poor, current evidence supports the conclusion that the benefits of universal iron supplementation outweigh its risks.

Please see related article: https://bmcmedicine.biomedcentral.com/articles/10.1186/s12916-018-1146-z.

Please see related article: https://bmcmedicine.biomedcentral.com/articles/10.1186/s12916-019-1376-8.

**Electronic supplementary material:**

The online version of this article (10.1186/s12916-019-1375-9) contains supplementary material, which is available to authorized users.

## Background

A recent cohort study in Papua New Guinean pregnant women reported an association between iron deficiency (ferritin concentration < 15 μg/L) and higher birth weight (by 230 g), particularly among primigravidae [1]. Despite this finding, the authors conclude that it is essential to provide both iron supplementation and effective malaria chemoprophylaxis during pregnancy. This intriguing report warrants close scrutiny since the result seems to contradict current guidelines by the World Health Organization (WHO) recommending daily oral iron supplementation as part of antenatal care to prevent maternal anaemia, puerperal sepsis, low birth weight and preterm birth [2].

The WHO recommendation was based on a meta-analysis of randomised trials in which antenatal iron supplementation was suggested to lead to a slight increase in birth weight (24 g, 95% CI − 3 g to 50 g) [3]. The magnitude of this effect is probably grossly underestimated because the meta-analysis was based on trials lacking a true placebo arm, and in which women with anaemia or iron deficiency were excluded, or were treated if they developed anaemia or iron deficiency. Indeed, the only placebo-controlled trial performed to date that enrolled and followed women with iron deficiency or mild and moderate anaemia was published after the WHO meta-analysis [4]. This study found that iron supplementation increased birth weight by 234 g (95% CI 116–351 g) in iron-deficient women, consistent with the fact that absorption and utilisation of supplemental iron is enhanced in iron deficiency. Thus, the Papua New Guinean results seem to be at variance with previous studies. What could explain this divergence?

## Antenatal iron supplementation and birth weight

**Fig. 1:**
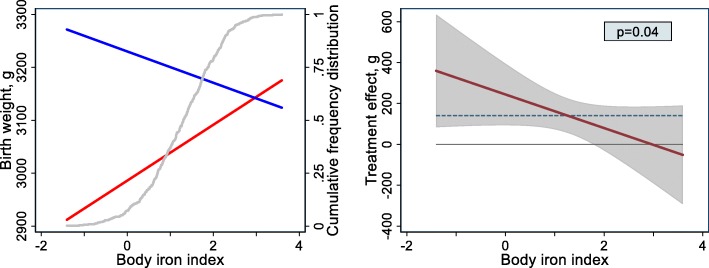
Effect of antenatal iron supplementation on neonatal birth weight in Kenya, by iron status at baseline. Iron status is indicated by body iron index, i.e. the natural logarithm of the ratio of plasma concentrations of ferritin (μg/L) and soluble transferrin receptor (mg/L), both adjusted for inflammation and *Plasmodium* infection (see text). Effects were adjusted for HIV infection and parity; other covariates were eliminated in the multiple fractional polynomial regression (*mfpi)* procedure. Based on analysis of 391 Kenyan women. *Left panel*: Associations between birth weight and body iron index for women who received supplementation with iron (blue line) or placebo (red line). The difference between these lines is the treatment effect (i.e. the difference in mean birth weight between the iron group and the placebo group, with the placebo group used as the reference) conditional to body iron index. The cumulative relative frequency distribution of the body iron index is indicated by the grey line (and right *Y*-axis); 95% of women had values in the range between − 0.55 and 2.82. *Right panels*: treatment effect as a function of body iron index, with corresponding 95% confidence bands and *p* value for interaction. The horizontal solid line indicates zero effect, whilst the horizontal dashed line indicates the intervention effect as measured in a regression model without covariates other than the intervention (140 g)

We re-analysed data from our placebo-controlled trial in pregnant Kenyan women [4] to assess to what extent iron status at baseline (i.e. second trimester of pregnancy) modified the magnitude of the effect of antenatal iron supplementation on neonatal birth weight (see Additional file 1 for details and statistical methods). Maternal iron status was assessed by plasma concentrations of ferritin and soluble transferrin receptor, which were adjusted for inflammation and *Plasmodium* infection. These markers were used to calculate the body iron index, i.e. the natural logarithm of the ratio of the adjusted ferritin concentration to the adjusted transferrin receptor concentration. This quantitative indicator has been shown to be linearly associated with the size of the body iron store in iron-replete individuals, and with the size of the functional deficit that would need to be corrected before iron could again be accumulated in the store in an iron-deficient individual. Multiple fractional polynomial regression analysis showed that the birth weight response to administered iron decreased with initial iron status (interaction *p* = 0.04; Fig. 1). In addition, in women who received iron supplementation, birth weight declined with iron status (Fig. 1, blue line).

In the Papua New Guinea study, iron deficiency was assessed at enrolment, at a mean gestational age of 25 weeks (SD 4.2 weeks). All women in the study were prescribed daily supplementation with iron and folic acid. Consistent with our findings in Kenyan women, iron-supplemented women who were initially iron deficient had newborns with a higher birth weight than their peers who were initially iron replete. What could explain this phenomenon?

In normal pregnancies, plasma volume expansion leads to a gradual reduction in haemoglobin concentration and haematocrit levels in the gestational age period between 7 and 36 weeks, despite concurrent increases in the total erythrocyte and haemoglobin mass [5]. This haemodilution is believed to increase oxygen delivery to tissues and organs by reducing blood viscosity and thus increasing blood velocity, particularly in the microcirculatory vessels of the maternal intravillous space. Thus, there is an optimum haematocrit level for oxygen delivery that is lower than haematocrit values found in healthy, non-pregnant women [6]. Haemodilution is vital for a healthy pregnancy, and analyses of large numbers of women of various races have shown that there is a pronounced U-shaped curve with the lowest risk of low birth weight and prematurity occurring well below the WHO definition of anaemia [7].

In iron-deficient women, haemodilution without an adequate supply of iron to the developing erythrocyte and haemoglobin mass will likely result in placental insufficiency, leading to low birth weight and prematurity. This may explain the negative association that we found between iron status and birthweight in women who were not supplemented with iron (Fig. 1, red line). Conversely, when iron deficiency is reversed by iron supplementation, birth weight is increased. With increasing iron status, however, the elevated haematocrit levels due to iron supplementation will increase blood viscosity. At some point, haematocrit levels will exceed the optimum value, resulting in a decreased delivery of oxygen (and other nutrients) and decreasing birth weight (Fig. 1, blue line). The crossing of red and blue lines in our figure suggests that iron supplementation can even lead to a reduced birth weight in women with high initial iron status, but this may have affected very few women (grey line) and supportive evidence is weak (see 95% confidence bands in right panel).

Because all women in the Papua New Guinea study were prescribed iron supplementation, it is impossible for its effect to be determined. Based on the evidence reviewed in the preceding sections, we speculate that birth weight of children born to women who were initially classified as iron deficient was increased by iron supplementation. Thus, the results from the trials and the Papua New Guinea study are not necessarily contradictory.

## Antenatal iron supplementation and malaria risk

There is ample evidence that iron supplementation predisposes towards the proliferation of pathogenic microorganisms. In vitro studies indicate that asexual blood stages of *P. falciparum* preferably invade and propagate in young erythrocytes, which transiently increase in numbers when iron supplements are administered to iron-deficient individuals [8, 9]. These findings may explain the transient increase in malaria risk that has been observed in association with iron supplementation in infants and preschool children [9]. In addition, the enhanced blood viscosity and decreased blood velocity ensuing from iron supplementation might conceivably increase the number of *P. falciparum*-parasitized erythrocytes that cytoadhere to the vascular endothelium [6], thus avoiding clearance by the spleen. Two large trials recently undertaken to evaluate the effect of antenatal iron supplementation on maternal *P. falciparum* infection (reviewed by [9]) showed no evidence of an increased risk. Iron supplementation may be safer in women than in children because women have higher levels of acquired immunity against *Plasmodium* infection. Even among women who received no intermittent preventive therapy, iron supplementation appeared to increase birth weight (by 167 g) without an apparent effect on *P. falciparum* infection risk [4].

## Conclusion

Randomised trials offer superior methodology and should be given higher priority in the hierarchy of evidence than cohort studies because they minimise the risk of selection bias. Benefits of universal iron supplementation in pregnancy are likely to vary with the prevalence of iron deficiency [9] and are thus context specific. In low- and middle-income countries, these benefits include increased neonatal birth weight and infant iron stores, reduced risk of severe maternal anaemia and maternal blood transfusion and premature delivery [9]. Restriction of iron supplementation to pregnant women who are screened and diagnosed with iron deficiency during antenatal clinics is not a feasible alternative to universal iron supplementation [9].

Antenatal malaria prevention not only results in direct health benefits to mothers and neonates, but has the additional advantage that elimination of inflammation associated with chronic *Plasmodium* infection may also improve the absorption of non-haem iron from supplements or dietary sources [10].

The efficacy and coverage of preventive measures against malaria in pregnancy are generally low. For example, among African countries that reported on intermittent preventive treatment in pregnancy in 2016, only 19% received the recommended minimum of three doses of intermittent preventive treatment [11]. It seems prudent to provide antenatal iron supplementation in conjunction with measures to prevent, diagnose and treat malaria. However, current evidence supports the conclusion that the benefits of universal iron supplementation outweigh the risks in conditions of high exposure to infectious diseases, even with poor coverage of preventive measures against malaria.

## Additional file

Additional file 1: Methods to assess the influence of maternal body iron status at baseline (i.e. second trimester of pregnancy) on the magnitude of the effect of antenatal iron supplementation on neonatal birth weight. (DOCX 19 kb)

## Data Availability

The datasets used for the current study are available from the corresponding author on reasonable request.
