# Supplementary material for: Antenatal iron supplementation and birth weight in conditions of high exposure to infectious diseases
Source: BMC Med. 2019 Jul 26;17:146. doi: 10.1186/s12916-019-1375-9 (PMC6659278; doi:10.1186/s12916-019-1375-9)
Supplement: Supplementary file 1 — Methods to assess the influence of maternal body iron status at baseline (i.e. second trimester of pregnancy) on the magnitude of the effect of antenatal iron supplementation on neonatal birth weight. (DOCX 19 kb) [file 12916_2019_1375_MOESM1_ESM.docx]

Additional file 1

In the report of our placebo-controlled trial in Kenyan women [1], we conducted subgroup analysis to assess the influence of baseline maternal iron status on the response in birth weight to antenatal iron supplementation. Iron status was defined as categories (replete, deficient, uncertain), depending on concentrations of ferritin and inflammatory markers. However, categorisation of continuous variables leads to loss of information, it is statistically inefficient, and results may depend strongly on the cut-off values selected. To avoid these shortcomings, we re-analysed these data using multiple fractional polynomial regression, which allows investigation of possible interactions between treatment and continuous covariates.

Maternal iron status at baseline was assessed using plasma concentrations of ferritin and soluble transferrin receptor (sTfR). Interpretation of these iron markers is complicated because they are influenced by infection and inflammation independently of iron status. To account for these troublesome effects, we used linear regression models proposed by the BRINDA group [2, 3] with some modifications: a) we used a Box-Cox power transformation of ferritin concentrations with $\lambda$ selected to yield zero skewness (*bcskew0* command in Stata); b) we included plasma *α*_1_-acid glycoprotein concentration and *Plasmodium* infection as independent variables (assessment by multiple fractional polynomial regression analysis and Bayesian Information Criterion showed no evidence that inclusion of plasma C-reactive protein concentration or power transformations of plasma *α*_1_-acid glycoprotein concentration improved model fit), and c) we used no reference value for *α*_1_-acid glycoprotein concentration. For sTfR concentration, we similarly used a Box-Cox transformation and included *Plasmodium* infection as an explanatory variable (there was no evidence that inclusion of plasma concentrations of C-reactive protein or *α*_1_-acid glycoprotein improved model fit). Plasma concentrations of ferritin and sTfR were re-expressed into natural units by back-transformation using the formula $y^{*}=\left( y\cdot\lambda+1 \right)^{\frac{1}{\lambda}}$. We used the results to calculate the body iron index, i.e. the natural logarithm of the ratio of the adjusted ferritin concentration to the adjusted transferrin receptor concentration. This indicator has been shown to be linearly associated with quantitative estimate of the size of the body iron store in iron-replete individuals, and with the size of the functional deﬁcit that would need to be corrected before iron could again be accumulated in the store in an iron-deﬁcient individual [4].

We used multiple fractional polynomial regression analysis to explore to what extent iron status at baseline modified the magnitude of the effect of iron supplementation on birth weight, anticipating that iron absorption and thus the response to administered iron would be larger in iron-deficient women than in their iron-replete peers. We used the *mfpi* procedure in Stata software [5, 6] with the ‘*flex*(3)’ specification to define the flexibility of the main effects and interaction models [7,8], adjusting for potentially influential maternal characteristics assessed at randomisation, i.e. haemoglobin concentration, age, body mass index, gestational age, parity, HIV infection and *Plasmodium* infection. We used a nominal significance level of 0·05 for selection of variables and power functions; selection of linear, first-degree or second-degree polynomials was based on the lowest value for Akaike’s information criterion. This procedure yielded a model for birth weight with linear terms for body iron index and its interaction with iron supplementation (p=0.04), whilst adjusting for HIV infection and parity (Figure in main text).

References

1. Mwangi MN, Roth JM, Smit MR, Trijsburg L, Mwangi AM, Demir AY, Wielders JP, Mens PF, Verweij JJ, Cox SE, Prentice AM, Brouwer ID, Savelkoul HF, Andang'o PE, Verhoef H. Effect of daily antenatal iron supplementation on Plasmodium infection in Kenyan women: a randomized clinical trial. JAMA 2015;314:1009–20.
2. Namaste SML, Rohner F, Huang J, Bhushan NL, Flores-Ayala R, Kupka R, Mei Z, Rawat R, Williams AM, Raiten DJ, et al. Adjusting ferritin concentrations for inflammation: Biomarkers Reflecting Inflammation and Nutritional Determinants of Anemia (BRINDA) project. Am J Clin Nutr. 2017;106(Suppl):359S–71S.
3. Rohner F, Namaste SML, Larson LM, Addo OY, Mei Z, Suchdev PS, Sakr Ashour FA, Rawat R, Raiten DJ, Northrop-Clewes CA. Adjusting soluble transferrin receptor concentrations for inflammation: Biomarkers Reflecting Inflammation and Nutritional Determinants of Anemia (BRINDA) project. Am J Clin Nutr. 2017;106(Suppl):372S–82S.
4. Skikne BS, Flowers CH, Cook JD. Serum transferrin receptor: a quantitative measure of tissue iron deficiency. Blood 1990;75:1870–76.
5. Royston P, Sauerbrei W. A new approach to modelling interactions between treatment and continuous covariates in clinical trials by using fractional polynomials. Stat Med. 2004;23:2509–25.
6. Royston P, Sauerbrei W. Two techniques for investigating interactions between treatment and continuous covariates in clinical trials. Stata J. 2009;9:230–51.
7. Royston P, Sauerbrei W. Interaction of treatment with a continuous variable: simulation study of significance level for several methods of analysis. Stat Med. 2013;32:3788–803.
8. Royston P, Sauerbrei W. Interaction of treatment with a continuous variable: simulation study of power for several methods of analysis. Stat Med. 2014;33:4695–708.
